# Discrepancies in prescribed medications as reported by patients, general practitioners, and community pharmacists in older patients with polypharmacy in primary care

**DOI:** 10.1186/s12875-026-03332-3

**Published:** 2026-04-30

**Authors:** Donna Bosch-Lenders, Henri E. J. H. ‘Jelle’ Stoffers, Bjorn Winkens, Mascha Twellaar, Wouter J.M. Hufen, Hugo P. van der Kuy, J. André Knottnerus, Marjan van den Akker

**Affiliations:** 1 Department of Family Medicine, Care and Public Health Research Institute (CAHPRI), Maastricht University, PO Box 616, Maastricht, MD 6200 The Netherlands; 2 Department of Methodology and Statistics, Care and Public Health Research Institute (CAHPRI), Maastricht University, PO Box 616, Maastricht, MD 6200 The Netherlands; 3 Department of General Practice, Amsterdam UMC, Vrije Universiteit Amsterdam, PO Box 7057, Amsterdam, DD 1100 The Netherlands; 4 Department of Hospital Pharmacy, Erasmus MC, University Medical Center Rotterdam, PO Box 2040, Rotterdam, GD 3015 The Netherlands; 5 Department of General Practice and Family Medicine, Medical Faculty OWL, Bielefeld University, Konsequenz 41c, Bielefeld, 33615 Germany; 6 Institute of General Practice, Goethe University, Theodor-Stern-Kai 7, Frankfurt am Main, 60590 Germany

**Keywords:** Polypharmacy, Primary care, General practitioner, Community pharmacist, Medication discrepancy, Medication reconciliation

## Abstract

**Background:**

Studies have shown considerable discrepancies between the medication lists reported by patients, physicians, and pharmacists.

**Objective:**

To investigate discrepancies in prescribed medications as reported by patients, general practitioners (GPs), and community pharmacists (CPs) in older patients with polypharmacy.

**Methods:**

Study design: Cross-sectional analysis. Setting: Twenty-four GP centres and 17 associated community pharmacies in the Netherlands. Data sources: Postal questionnaires, home visits, and the electronic patient record systems of the GPs and CPs. We defined a medication discrepancy as any inconsistency between the patient’s, GP’s, or CP’s medication list. For fixed medications, we included inconsistencies in the medication’s name, dose, or frequency; for variably used medications, we included only inconsistencies in the medication’s name. Logistic regression analyses and GEE were performed to identify discrepancy-related patient and medication characteristics.

**Results:**

Data from 751 patients (mean age 72.9 years (SD 7.6), 47.3% female) using 5989 prescribed medications (average 8, range 3–19) were analysed. The overall patient-GP-CP discrepancy was 71.9% at the patient level and 21.8% at the medication level. Statistically significant associations with discrepancy included various medication characteristics, e.g. any additional medication (OR 1.45, 95% CI 1.33–1.59) and variable medication usage (varying ORs 1.61–5.78), and the patient characteristic ‘Living in a residential care home’ (OR 0.42, 95% CI 0.18–0.997).

**Conclusion:**

We found a high patient-GP-CP discrepancy, with a 72% discrepancy in prescribed medication at the patient level and a 22% discrepancy at the medication level. ‘Living in a residential care home’ reduced the chance of medication discrepancy, whereas medication characteristics, such as variable medication usage, increased it.

**Trial registration:**

NLOMON32535 (https://onderzoekmetmensen.nl/en/trial/32535 registered 15th December 2009).

**Supplementary Information:**

The online version contains supplementary material available at 10.1186/s12875-026-03332-3.

## Background

Polypharmacy is usually defined as the chronic use of five or more medications [1]. Multimorbidity is the leading cause since multiple diseases require multiple treatments. However, polypharmacy can cause collateral damage, such as a high treatment burden and decreased quality of life [2], hospitalisation, and mortality due to adverse medication events and interactions [3]. Despite limited evidence on patient-related outcomes, regular medication reviews are commonly accepted and recommended, helping improve medication appropriateness [4]. Guidelines for medication reviews exist in various countries, including the Netherlands [5, 6]. In primary care, the key actors are the patient, the general practitioner (GP), and the community pharmacist (CP).

An essential part of a medication review is a comprehensive and up-to-date overview of prescribed medications. Ideally, there should be no discrepancy between key actors regarding the medications the patient takes. However, studies have shown considerable discrepancy between the medication lists reported by patients, physicians, and pharmacists [7–10]. This can also jeopardise medication safety because of the risk of overtreatment when medication is prescribed more than once by different care providers.

The ‘Polypharmacy Intervention Limburg’ (PIL) study - a Dutch cluster randomised controlled trial – evaluated the effects of a comprehensive medication review (including over-the-counter (OTC) medicines) on quality of life and medication use in patients aged 60 + years with polypharmacy [11]. A distinctive feature of this study was the home visits, which provided us with the most accurate possible overview of the medications patients reported taking. In a preliminary analysis of a part of the PIL dataset, we found only a modest concordance between the numbers of prescribed medications as reported by the patient, the GP and the CP, respectively [12]. The aim of the current analysis, using baseline data from the PIL study, was to assess the reported medication use of older patients with polypharmacy and to investigate discrepancies between the prescribed medication lists reported by the patient, the GP, and the CP. Our goal was to identify clues to prevent medication list discrepancies and thereby improve GP and CP medication management and patient medication safety.

## Methods

### Study design

The current study uses cross-sectional baseline data from the PIL study [11, 13], a two-armed cluster-randomised controlled trial. It used a stepped-wedge design with unexposed (usual care) and exposed (intervention: a comprehensive medication review) observations. The baseline data were collected between October 2010 and February 2012 [11].

### Recruitment of participants

#### Healthcare professionals

We invited all general practices in South Limburg, the Netherlands, by letter to participate in the PIL study. Twenty-four general practice centres (43 GPs, 21 practice nurses) - including eight single-handed practices, eight partnership practices, and eight medical centres - agreed to participate. In addition, seventeen community pharmacies associated with the joining GP centres participated. The fixed combinations of GP practices and pharmacies received joint training.

#### Patients

First, the CPs identified patients aged 60 years or over with polypharmacy - defined as chronic use, i.e. more than three months per year of five or more prescribed medications - according to the pharmacy’s information system. Then, the GPs identified ineligible patients based on the following criteria: an estimated life expectancy of less than one year, incompetence in making their own decisions as assessed by the GP, or inability to read or write. From the remaining pool of eligible patients, each practice invited a fixed (target) number of patients, depending on the list size - i.e. the size of the practice patient population enrolled in a practice (list size up to 2000: target 30 patients; list size 2000–5000: target 40 patients; list size > 5000: target 60 patients) - using a randomly ordered list of eligible patients. A trained practice nurse contacted patients by phone and provided information about the study. Interested patients received a letter with additional information and an informed consent form [11]. Participating GPs and CPs received €25 for each patient included in the study for whom they performed a medication review. Additionally, GPs received €30 per patient for the practice nurses’ home visits. Participating patients did not receive any kind of compensation.

### Data collection

For the study reported here, we used cross-sectional baseline data collected between October 2010 and February 2012, i.e. before the intervention, when all patients received care as usual. Data were drawn from four independent sources as outlined in Table 1 [11].

**Table 1:** Baseline data of the PIL study [11, 13] used in the current analysis

Data provided by patients	
*Patient questionnaires (by post)*	• Postal code, sex (male/female), date and country of birth, partner (yes/no)
	• Level of education: none (no education), low (primary education, preparatory secondary vocational education), intermediate (vocational education, secondary education) or high (higher professional education or university)
	• Living situation: living independently alone, living independently with other(s), or living in a residential care home
Home visits by practice nurse*	• Current medication use as reported by the patient
	o Prescribed medications
	o Over-the-counter (OTC) medicines
Data provided by general practitioners (extracted from the GP’s EPR system)	• Current list of prescribed medications
Data provided by community pharmacists (extracted from the pharmacy’s EPR system)	• Current list of prescribed medications

*EPR* Electronic patient record

* Practice nurses were trained during a 3-hour workshop. They were explicitly instructed not to use a practice-based medication list during the home visits, nor give patients suggestions or make interpretations regarding the medication list.

### Variables and categories

#### Patient characteristics

Sex (male/female); age (60–69, 70–79, ≥ 80 years); partner (yes, no); level of education (intermediate or high, no or low); living situation (residential care home, independently alone, independently with other(s)); number of prescribed medications, as reported by the patient (≤ 5, 6–7, 8–9 or ≥ 10); number of OTC medicines/patient (0, ≥ 1). (Note: Although the inclusion criterion was five or more medications according to the pharmacy’s information system at the time of inclusion, some patients reported fewer than five prescribed medications during the home interview).

#### Prescribed medication characteristics

- *Prescribed medication groups*. The prescribed medications were categorised into eight clinical categories: ‘Cardiovascular’, ‘Digestive tract’, ‘Diabetes’, ‘Respiratory tract’, ‘Analgesics’, ‘Psychotropic’, ‘Other, oral’ and ‘Other, topical’. These categories resulted from previous discussions among regional groups of GPs affiliated with the Department of Family Medicine at Maastricht University on medication reviews. All the medications were assigned an ATC (Anatomical Therapeutic Chemical) code [14, 15].
- *Medication usage*: ‘fixed’ or ‘variable’. ‘Fixed’ medications have a defined dose and frequency per time unit [14]; all other medications were determined to be ‘variable’. If the official description of the medication (ATC code) did not indicate a dose and frequency for fixed usage [14], the patient’s reported usage determined whether the usage was categorised as fixed or variable (Details in Table 2).
- *Medication mode* comprised six categories: ‘Daily’, ‘Periodic’, ‘Daily but inappropriate’, ‘On-demand, conditional’, ‘On-demand, personal’, and ‘On-demand, inappropriate’ (Details in Table 2).
- *Medication administration* was described in nine categories: ‘Oral’, ‘Injections’, ‘Inhalation (respiratory)’, ‘Oromucosal’, ‘Patch’, ‘Enema’, ‘Suppository’, ‘Eye/ear drops’ and ‘Dermal’.

**Table 2:** Medication usage (fixed/variable) and medication mode categories

Medication usage	Medication mode - description	Medication mode - short name	Examples
Fixed	Chronically indicated, daily use (incl. daily used eye drops with fixed use)	Daily	*Antihypertensive medications*,* Timolol eye drops for glaucoma*
	Chronically indicated, with planned periodic use.	Periodic	*Bisphosphonates*
	Daily use despite no or inappropriate indication	Daily but inappropriate (wrong indication, expired indication)	*Daily use of sedatives*
Variable	Chronically indicated, but conditional daily or periodic use (‘if … then …’)	On-demand, conditional	*Painkillers in osteoarthritis*, PPI in reflux oesophagitis*,* Laxatives (constipation)*
	Chronically indicated, but varying consumption due to varying time period or varying quantity/dose, at the patient’s personal discretion	On-demand, personal	*Ointments (e.g. for prurigo)*,* Isosorbide dinitrate (angina pectoris)*,* Salbutamol Inhalation (COPD symptoms)*,* Eye drops (allergic rhinitis)*
	On-demand use despite no or inappropriate indication	On-demand, inappropriate (wrong indication, expired indication)	*Chronic laxative use*,* PPIs for stomach pain*,* codeine for cough*

* The examples in Table 2 reflect correct use during the study period. Currently, the insights for Proton Pump Inhibitors (PPIs) have changed, namely to reduce the dosage as quickly as possible and eventually stop the medication

Appropriateness was based on existing clinical practice guidelines for GPs and CPs, as well as pharmacotherapeutic guidelines available at the time [14].

#### OTC medicines

OTC medicines were registered as reported by each patient during the practice nurse’s home visit. Details can be found in the Supplement (Table S1), including data on potentially dangerous OTC medicines (oral nonsteroidal anti-inflammatory drugs (NSAIDs) and antimycotics (CYP2C9 Inhibitors)) [14, 16].

#### Discrepancy between patients, GPs, or CPs on prescribed medications

We defined medication discrepancy as any inconsistency between the patient’s, GP’s or CP’s medication list. For fixed medication, we included inconsistencies in the medication’s name (ATC-code), dose or frequency (‘NDF criterion’); for variable medication, we included only inconsistencies in the medication’s name (ATC-code) (‘N criterion’) to prevent exaggerating the number of discrepancies. With variably used medications, actual discrepancies in dose or frequency — in the sense of errors or inaccuracies — are hard to identify validly.

### Statistical analyses

SPSS was used for all analyses (IBM Corp. Released 2016. IBM SPSS Statistics for Windows, Version 24.0. Armonk, NY: IBM). Descriptive analyses were performed on patient characteristics, characteristics of prescribed medications and OTC medicines, and medication list discrepancies between the patient, the GP, or the CP. Analyses were performed at the medication level (i.e., using all medications as the study sample) and at the patient level (using all patients as the study sample). The results are presented as frequencies and percentages, or as means with standard deviations (SD) and ranges.

To find clues to avoid medication list discrepancies, we performed logistic regression analyses to assess the independent associations between ‘discrepancy between patients, GPs or CPs’ (dependent variable) and patient characteristics, prescribed medication categories, and medication mode (independent variables). To adjust for the possible clustering effects, additional generalised estimating equation (GEE) analyses were performed. The GEE analysis method was used to adjust for the possible clustering effects (patients clustered within GP practices). An exchangeable working correlation structure was used to account for the intra-class correlation between outcomes from the patients within the same GP practice. The GEE method was used instead of hierarchical methods (e.g., a logistic mixed-effects model) because we were not interested in results from individual GP practices but rather in the population average across all GP practices. Odds ratios (ORs) with 95% confidence intervals (CIs) are reported. A two-sided p-value of ≤ 0.05 was considered statistically significant. We did not impute missing data because the numbers were relatively low; for most variables in the discrepancy analyses, the proportion of missing values was around 2–3% (see Table S3). For the logistic regression and GEE analyses, 3–6% of patients were excluded due to missing one or more variables.

This study presents a cross-sectional analysis of baseline data from the PIL cluster randomised controlled trial. Therefore, the study is reported in accordance with the STROBE guidelines for observational studies (see appendix).

## Results

Our trial included 772 patients. One of the participating practices, with 21 included patients, did not provide the required medication information. Therefore, we included 751 patients in the current analysis.

### Patient characteristics

Table 3 presents the characteristics of the 751 patients with polypharmacy (mean age 72.9 years, 47.3% female).

**Table 3:** Patient characteristics of 751 older patients with polypharmacy in primary care

Patient characteristic		*n* (% or SD)
Gender	Female (%)	355 (47.3)
	Male (%)	396 (52.7)
Age, years	Mean (SD)	72.9 (7.6)
	Range	60–94
	60–69 (%)	292 (38.9)
	70–79 (%)	293 (39.0)
	≥ 80 (%)	166 (22.1)
Level of education *	Intermediate or high (%)	213 (28.4)
	No or low (%)	526 (70.0)
	Missing information (%)	12 (1.6)
Living situation	Independent with other(s) (%)	494 (65.8)
	Independent alone (%)	207 (27.6)
	Residential care home*** (%)	37 (4.9)
	Missing information (%)	13 (1.7)
Partner	Yes (%)	493 (65.6)
	No (%)	246 (32.8)
	Missing information (%)	12 (1.6)
Number of prescribed medications per patient **	Mean (SD)	8.0 (2.6)
	Range	3–19
	1–5 (%)	114 (15.2)
	6–7 (%)	261 (34.8)
	8–9 (%)	187 (24.9)
	≥ 10 (%)	189 (25.2)
Number of OTC medicines per patient **	Mean (SD)	1.1 (1.2)
	Range	0–6
	0 (%)	294 (39.1)
	≥ 1 (%)	457 (60.9)

*OTC* Over-the-counter, *SD* Standard deviation

* Level of education: definitions in Table 1

** According to the patient (see Methods)

*** residential centre for older people; support only by ‘residential care workers’, i.e. without nursing or medical responsibilities

### Medication characteristics

A total of 457 patients (70.9%) in the study population reported taking OTC medicines (*N* = 822). On average, patients reported taking one OTC medicine (range: 0–6). As for potentially dangerous OTC medicines, oral NSAIDs (*n* = 10) were taken by nine patients (2.0%), and one patient took the antimycotic miconazole (*n* = 2) (Details in Supplement Table S1).

Table 4 shows the prescribed medications (*N* = 5989) for patients (*N* = 751) who used at least one medication in the described category. On average, patients reported taking eight prescribed medications (range 3–19). Virtually all (97.3%) studied patients were taking cardiovascular medications, accounting for 53.4% of all prescribed medications. Of all prescribed medications, medication usage was fixed in 69.6% versus variable in 30.4%, and 65.4% were reported to be taken daily (medication mode). At least one variable medication was used by 635 (84.6%) patients.

**Table 4:** Prescribed medication characteristics in older patients with polypharmacy in primary care who use at least one medication in the described category

Prescribed medication characteristics		Medication level (*N* = 5989)		Patient level (*N* = 751)		Medications/patient	
Prescribed medication categories		Number of prescribed medications	% of all prescribed med.	Number of patients who use ≥ 1 med. in the category	% of all patients	Mean number of medications per patient	Range in number of med. per patient
All		5989	100.0	751	100.0	8.0	3–19
Cardiovascular		3197	53.4	731	97.3	4.4	1–10
Digestive tract		504	8.4	405	53.9	1.2	1–4
Diabetes		424	7.1	262	34.9	1.6	1–4
Respiratory tract		386	6.4	207	27.6	1.9	1–7
Analgesics		245	4.1	204	27.2	1.2	1–4
Psychotropic		229	3.8	189	25.2	1.2	1–4
Other, oral		685	11.4	396	52.7	1.7	1–10
Other, topical		319	5.3	193	25.7	1.7	1–7
Medication usage	Medication mode						
Fixed*		4170	69.6	751	100	5.6	1–12
	Daily^	3919	65.4	746	99.3	5.3	1–12
	Periodic^^	131	2.2	119	15.8	1.1	1–3
	Daily but inappropriate^^^	120	2.0	108	14.4	1.1	1–3
Variable**		1819	30.4	635	84.6	2.9	1–12
	On-demand, conditional^˅^	782	13.1	474	63.1	1.6	1–8
	On-demand, personal^˅˅^	984	16.4	469	62.5	2.1	1–8
	On-demand, inappropriate^˅˅˅^	53	0.9	52	6.9	1.0	1–2

*Fixed medication usage: Defined dose and frequency per time unit; Variable medication usage

** no defined dose and/or frequency per time unit. Medication mode

^^^ chronically indicated, daily use

^^ chronically indicated, with planned periodic use

^^^ daily use despite no or inappropriate indication

^˅^ chronically indicated, but conditional daily or periodic use

^˅˅^ chronically indicated, but varying consumption

^˅˅˅^ on-demand use despite no or inappropriate indication (For detailed definitions of and examples on Medication usage (fixed/variable) and Medication mode, see Table 2)

### Discrepancy between patients, GPs, or CPs on prescribed medications

In the discrepancy analyses, 12 injections administered by the GP were excluded. In addition, we had missing data for 135 medications (2.3%), leaving 5842 medications for analysis. The percentage of missing data was similar across medication categories (range 2.0-3.1%). More details are shown in the Supplement (Tables S2 and S3).

Table 5 shows that there were inconsistencies between patients, GPs, or CPs in 21.8% of all prescribed medications (using the NDF criterion for fixed medication usage and the N-criterion for variable medication usage). At the patient level, the overall discrepancy between patients, GPs or CPs regarding prescribed medications was 71.9%. The most common discrepancies were medications only mentioned by the patient (32.4% of all discrepancies), medications not registered by the GP (19.5% of all discrepancies), medications not registered by the CP (14.1% of all discrepancies), and dosage differences (34.0% of all discrepancies).

**Table 5:** Discrepancy between patients, general practitioners (GPs) or community pharmacists (CPs) for prescribed medication categories, medication mode and medication administration categories using criteria^*^ considering fixed^#^ or variable medication^&^ usage

Prescribed medication characteristics	Medication level		Patient level		Medications/patient	
	Number (%) of medications per category	Number (%) of medications with discrepancy* between Patient, GP, or CP	Number (%) of patients with ≥ 1 medication in the category	Number (%) of patients with discrepancy* between patient, GP, or CP for *any* medication in that category	Mean number of medications per patient for that category	Mean number (%) of medications with discrepancy* between patient, GP, or CP per patient for that category
All **	**5842 (100)**	**1307 (21.8)**	**732 (100)**	**526 (71.9)**	**7.98**	**1.78 (22.3))**
Prescribed medication category						
Cardiovascular	3133 (100)	476 (15.2)	713 (100)	304 (42.6)	4.39	0.66 (15.0)
Digestive tract	492 (100)	114 (23.2)	395 (100)	98 (24.8)	1.25	0.29 (23.2)
Diabetes	415 (100)	72 (17.3)	256 (100)	56 (21.9)	1.62	0.28 (17.3)
Respiratory tract	377 (100)	129 (34.2)	201 (100)	101 (50.2)	1.88	0.85 (34.6)
Analgesics	240 (100)	106 (44.2)	200 (100)	91 (45.5)	1.20	0.53 (44.2)
Psychotropic	222 (100)	75 (33.8)	183 (100)	70 (38.3)	1.21	0.41 (33.9)
Other, oral	652 (100)	180 (27.6)	384 (100)	145 (37.8)	1.70	0.47 (27.6)
Other, topical	311 (100)	155 (49.8)	187 (100)	96 (51.3)	1.66	0.83 (50.0)
Medication usage & Medication mode						
Fixed usage, all	4076 (100)	633 (15.5)	732 (100)	375 (51.2)	5.55	0.85 (15.3)
• Daily^	3835 (100)	567 (14.8)	728 (100)	398 (54.7)	5.27	0.78 (14.8)
• Periodic^^	126 (100)	31 (24.6)	115 (100)	85 (73.9)	1.10	0.27 (24.5)
• Daily, inappropriate^^^	115 (100)	35 (30.4)	104 (100)	71 (68.3)	1.11	0.34 (30.6)
Variable usage, all	1766 (100)	674 (38.2)	618 (100)	259 (41.9)	2.85	1.08 (37.9)
• On-demand, conditional ^˅^	762 (100)	241 (31.6)	460 (100)	284 (61.7)	1.66	0.53 (31.9)
• On-demand, personal ^˅˅^	951 (100)	403 (42.4)	453 (100)	193 (42.6)	2.10	0.89 (42.4)
• On-demand, inappropriate ^˅˅˅^	53 (100)	30 (56.6)	52 (100)	22 (42.3)	1.02	0.58 (56.9)
Medication Administration category						
Oral	4892 (100)	902 (18.4)	732 (100)	285 (38.9)	6.68	1.23 (18.4)
Non-oral, all	950 (100)	405 (42.6)	451 (100)	195 (43.2)	2.11	0.90 (42.7)
• Enema	4 (100)	1 (25.0)	4 (100)	3 (75.0)	1.00	0.25 (25.0)
• Inhalation (resp.)	316 (100)	96 (30.4)	173 (100)	97 (56.1)	1.82	0.55 (30.2)
• Patch	27 (100)	9 (33.3)	27 (100)	18 (66.7)	1.00	0.33 (33.0)
• Eye/ear drops	154 (100)	53 (24.4)	113 (100)	72 (63.7)	1.36	0.47 (34.6)
• Injections	152 (100)	57 (37.5)	112 (100)	69 (61.6)	1.36	0.51 (37.5)
• Oromucosal	127 (100)	89 (43.0)	121 (100)	44 (36.4)	1.05	0.66 (62.9)
• Dermal	166 (100)	106 (63.9)	98 (100)	31 (31.6)	1.69	1.08 (63.9)
• Suppository	4 (100)	3 (75.0)	4 (100)	1 (25.0)	1.00	0.75 (75.0)

* Discrepancy criteria:

For fixed medication usage: discrepancy on either drug name-dose-frequency (NDF-criterion)

or variable medication usage: discrepancy on drug name only (N-criterion)

In Supplement Table S4, we present the results of applying the NDF and N-criterion to all the medications, i.e., without distinguishing between fixed and variable usage

** 19 patients with missing data; 147 medications with missing data. Excluded (injections) and missing medications are explained in Supplement Tables S2, S3, and S4

^#^ Fixed medication usage: defined dose and frequency per time unit

^&^ Variable medication usage: no defined dose and/or frequency per time unit

Medication mode:

^^^ chronically indicated, daily use

^^^^ chronically indicated, with planned periodic use

^^^^^ daily use despite no or inappropriate indication

^˅^ chronically indicated, but conditional daily or periodic use

^˅˅^ chronically indicated, but varying consumption

^˅˅˅^ on-demand use despite no or inappropriate indication

(For detailed definitions of and examples on Medication usage (fixed/variable) and Medication mode, see Table 2)

#### Prescribed medication categories

At the patient level the largest category, Cardiovascular medication (713 patients), showed a discrepancy of 42.6%. There was relatively little discrepancy for patients using Diabetes or Digestive tract medications. At the medication level Cardiovascular (discrepancy 15.2%) and Diabetes medications (discrepancy 17.3%) scored relatively well (below average), whereas all other medication groups scored unfavourably (above average) (Details in Table 5).

#### Medication mode and medication usage 

At the patient level the largest category, Daily medication (728 patients), showed a discrepancy of 45.3%. Patients with ‘on-demand, personal’ medication scored the worst. At the medication level fixed usage showed a smaller discrepancy than variable usage (15.5% vs. 38.2% discrepancy); only daily used medications scored relatively well (below average, 14.8% discrepancy). Medications taken on demand with an inappropriate indication scored unfavourably (56.6% discrepancy) (Details in Table 5).

#### Medication administration 

Patients used seven oral medications, on average (range 1–15); discrepancy at the patient level was 61.1%. At the medication level oral medications scored favourably (below average, 18.4% discrepancy). Discrepancy percentages for oromucosally (63.0%) and dermally (63.9%) administered medications were high (Details in Table 5).

### Factors associated with discrepancies between patients, GPs, or CPs on prescribed medications

#### Patient characteristics 

Each additional prescribed medication a patient reported taking increased the chance of discrepancy (OR 1.45, 95% CI 1.33–1.59). There was a lower chance of discrepancy among patients living in a residential care home than among those living independently alone (OR 0.43, 95% CI 0.18–0.997). The other patient characteristics had no statistically significant associations with medication discrepancy. Logistic regression analysis and GEE resulted in similar results (Details in Table 6).

**Table 6:** Logistic regression and generalised estimating equations (GEE) analysis of discrepancy between patients, general practitioners (GPs) or community pharmacists (CPs) on prescribed medications for patient characteristics *

Patient characteristics		Number (%)	Logistic regression		GEE (ICC^§^ = 0.0049)	
			*p*	Adjusted Odds Ratio (OR) *** (95%CI)	*p*	Adjusted Odds Ratio (OR) *** (95%CI)
Sex	Male	380 (54.1)		1		1
	Female	323 (45.9)	0.763	1.061 (0.721–1.561)	0.731	1.063 (0.751–1.505)
Age (years)	60–69	282 (40.1)		1		1
	70–79	273 (38.9)	0.857	1.037 (0.700-1.534)	0.933	1.014 (0.735–1.399)
	≥ 80	148 (21.0)	0.506	1.191 (0.712–1.992)	0.492	1.170 (0.747–1.831)
Level of education	Low-intermediate	499 (70.9)		1		1
	High	204 (29.1)	0.086	1.440 (0.950–2.184)	0.091	1.426 (0.945–2.153)
Living situation	Independent alone	191 (27.2)		1		1
	Independent with other(s)	479 (68.1)	0.686	0.857 (0.405–1.814)	0.635	0.844 (0.419–1.701)
	Residential care home	33 (4.7)	0.049	0.425 (0.181–0.997)	0.012	0.436 (0.229–0.831)
Partner status	Partner	475 (67.6)		1		1
	No partner	228 (32.4)	0.657	1.182 (0.565–2.474)	0.636	1.174 (0.605–2.278)
Number of prescribed medications **		703 (100)	< 0.001	1.453 (1.327–1.591)	< 0.001	1.456 (1.338–1.583)
Over-the-counter (OTC) medicines	No OTC medicines	275 (39.1)		1		1
	OTC medicines	428 (60.9)	0.981	1.004 (0.702–1.437)	0.977	1.005 (0.709–1.424)

^§^ ICC = intra-class correlation, i.e., the correlation between the outcomes of patients within the same GP practice

* *N* = 703 patients; 48 patients with missing data

** Range 3–19 medications, average 8 medications (see Table 3)

*** Adjusted ORs for discrepancy on prescribed medication obtained from multiple logistic regression analyses, including gender, age, level of education, living situation, partner status, OTC medicines (no/yes) and number of prescribed medications

#### Prescribed medication categories 

Each additional cardiovascular medication a patient reported taking significantly increased the chance of discrepancy between the patient, GP, and CP on these medications (OR 1.23, 95% CI 1.10–1.37). Except for diabetes and digestive tract medication, this was similar for other medication categories (Details in Table 7).

**Table 7:** Logistic regression and generalised estimating equations (GEE) analysis of discrepancy between patients, general practitioners (GPs) or community pharmacists (CPs) on prescribed medications for prescribed medication categories and medication mode

Prescribed medication characteristics	Category	Number of patients ^#^	Logistic regression		GEE	
			*p*	Adjusted Odds Ratio (OR) (95%CI) **	*p*	Adjusted Odds Ratio (OR) (95%CI) **
Prescribed medication category					ICC^§^= 0.0086	
Cardiovascular ^%^	Number	732	< 0.001	1.228 (1.101–1.370)	< 0.001	1.234 (1.100-1.384)
Digestive tract *	None	337		1		1
	≥ 1	395	0.086	1.377 (0.956–1.984)	0.080	1.373 (0.962–1.958)
Diabetes *	None	476		1		1
	1	122	0.229	1.350 (0.828–2.199)	0.136	1.361 (0.908–2.041)
	≥ 2	134	0.232	1.331 (0.833–2.128)	0.151	1.339 (0.899–1.995)
Respiratory tract *	None	531		1		1
	1	95	0.066	1.722 (0.964–3.076)	0.060	1.699 (0.978–2.951)
	≥ 2	106	< 0.001	4.306 (2.258–8.215)	< 0.001	4.300 (2.363–7.827)
Analgesics *	None	532		1		1
	≥ 1	200	0.012	1.775 (1.136–2.773)	0.006	1.771 (1.177–2.665)
Psychotropic *	None	549		1		1
	≥ 1	183	0.002	2.064 (1.299–3.279)	0.009	2.119 (1.206–3.721)
Other, oral *	None	348		1		1
	1	217	0.005	1.807 (1.199–2.723)	0.005	1.832 (1.201–2.793)
	≥ 2	167	< 0.001	3.085 (1.851–5.142)	< 0.001	3.131 (1.970–4.977)
Other, topical *	None	545		1		1
	≥ 1	187	< 0.001	4.192 (2.521–6.969)	< 0.001	4.188 (2.574–6.816)
Medication mode^&^					ICC^§^= 0.0092	
Daily ^^^	Number	732	0.007	1.152 (1.039–1.277)	0.010	1.160 (1.035–1.299)
Periodic*^^^^	None	617		1		1
	≥ 1	115	0.033	1.819 (1.050–3.152)	0.070	1.813 (0.953–3.446)
Daily but inappropriate * ^^^^^	None	628		1		1
	≥ 1	104	0.115	1.572 (0.896–2.757)	0.264	1.601 (0.701–3.652)
On-demand, conditional * ^˅^	None	272		1		1
	1	248	0.019	1.617 (1.084–2.412)	0.062	1.612 (0.975–2.665)
	≥ 2	212	< 0.001	2.554 (1.574–4.147)	< 0.001	2.577 (1.640–4.050)
On-demand, personal * ^˅˅^	None	279		1		1
	1	197	< 0.001	2.086 (1.380–3.153)	< 0.001	2.088 (1.385–3.147)
	≥ 2	256	< 0.001	4.993 (3.199–7.792)	< 0.001	4.944 (3.087–7.918)
On-demand, inappropriate * ^˅˅˅^	None	680		1		1
	≥ 1	52	0.001	5.682 (1.959–16.478)	0.002	5.725 (1.922–17.053)

^§^ ICC = intra-class correlation, i.e., the correlation between the outcomes of patients within the same GP practice

^#^*N* = 732 patients; 19 patients with missing data

* Categorical: reference category = 0 prescriptions in that medication category

** Adjusted ORs for discrepancy on prescribed medication obtained from multiple logistic regression analyses, with all medication categories as independent variables in the model

^%^ Since cardiovascular medications were prescribed to all patients (mean of 4.4 medications/patient, range 1–10), we analysed them as a continuous variable

^^^ chronically indicated, daily use. Since chronically indicated daily-use medications were prescribed to all patients (mean 5.3 medications/patient, range 1–12), we analysed them as a continuous variable

^^^^ chronically indicated, with planned periodic use

^^^^^ daily use despite no or inappropriate indication

^˅^ chronically indicated, but conditional daily or periodic use

^˅˅^ chronically indicated, but varying consumption

^˅˅˅^ on-demand use despite no or inappropriate indication

(For detailed definitions of and examples on Medication usage (fixed/variable) and Medication mode, see Table 2)

#### Medication mode 

Each additional chronically indicated daily used medication significantly increased the chance of discrepancy between the patient, GP, and CP in this medication mode (OR 1.15,95% CI 1.04–1.28). Other fixed medication modes did not show statistical significance after adjusting for patient clustering, but the ‘on-demand’ modes did (Details in Table 7).

## Discussion

The overall patient-GP-CP discrepancy on prescribed medication was 72% at the patient level and 22% at the medication level. ‘Living in a residential care home’ reduced the chance of discrepancy, whereas various medication characteristics, e.g. any additional medication and variable medication usage, increased it.

### Strengths and limitations

This is the first study to compare patient-reported medication lists with those recorded in GPs’ and CPs’ electronic patient records. We introduced the concepts of fixed vs. variable medication usage and medication mode (six categories), which proved helpful in providing greater insight into medication discrepancies between patients, GPs or CPs. The study sample was large and representative. Our patient-oriented approach ensured the most reliable data on patients’ medication: trained practice nurses conducted home visits to document patients’ actual medications. Nevertheless, our study does not allow conclusions about actual medication intake.

A possible limitation of this study is that its data were collected between 2010 and 2012. Since then, we have identified developments in the Dutch healthcare system that could influence the prescription (by GPs and specialists), delivery (by CPs) and use (by patients) of medication as well as the communication about medication by the three actors. These developments are described in detail in the Supplement, Box S5. We conclude that the identified developments may increase the chance that, currently, medication will be more often discussed between GPs, pharmacists, and patients. However, a common digital medication prescribing and dispensing system for doctors and pharmacists (‘MP 9’, see Supplement, Box 5) is still being developed and has not yet been implemented. Therefore, the discrepancies in medication between GPs, CPs and patients may have decreased, but there is little reason to assume that this change is substantial.

### Interpretation of the findings

#### Generalisability of the results

Despite regional differences in dominant health insurance companies, Dutch healthcare, including primary care, is organised comparably throughout the country. The same applies to the forms of collaboration between GPs and pharmacists. We recruited a variety of GP centres - including single-handed practices (33%; the Netherlands in 2012: 44%, in 2024: 16%) [17], partnership practices, and medical centres – and their main associated community pharmacies. We have no reason to assume that the variation in the degree of existing collaboration between the GPs and pharmacists participating in the study - e.g. in the form of regular ‘pharmacotherapeutic consultation sessions’ (see Supplement, Box 5) - will have been different from the rest of the Netherlands. Although the primary process among patient, GP and pharmacist is similar across countries, we hesitate to extrapolate our findings to other countries or healthcare systems. The degree of structured collaboration between GPs and CPs, as well as the degree of integration of medical information systems within the healthcare system, will influence the risk of discrepancies in medication lists between patients, GPs, or pharmacists.

#### Medication list discrepancy

However, we found a few studies from other countries, comparing patient-reported medication usage to that of doctors or pharmacists. Norwegian research evaluating the discrepancies between the medication lists of GPs, home care services and the CPs, found discrepancies at the medication level between the three healthcare services of 40% and 23% (baseline data control and intervention groups, respectively) [18]. A Swedish study reported comparable results when comparing pharmacy medication lists with patient-reported medications: 78% of patients mentioned discrepancies or missing prescriptions. At the medication level, they reported a 28% discrepancy [19]. Our results (not shown) for two-actor discrepancy at the medication level were 23% (patient-GP) and 20% (patient-CP), applying the NDF (name-dose-frequency) criterion to all medications. Summarising these and our studies leads to the conclusion that in primary care settings, discrepancy between patients and health professionals regarding prescribed medications is 70–80% at the patient level and 20–40% at the medication level.

Two non-primary care studies examining medication discrepancy at the time of hospital admission compared the best possible medication history (a combination of medical files, “brown bag”, community care prescriptions and patient interviews) with hospital records [9, 10]. These studies reported fewer discrepancies at the patient level (62% [9] and 50% [10]), but similar results (18% discrepancy) at the medication level [9, 10].

####  Factors influencing medication discrepancy 

A systematic review analysed factors associated with medication discrepancies at the time of hospital admission, comparing pharmacist-assisted medication reconciliation (including patient reports) to hospital information (hospital medical records, physician medication histories or the medical team’s medication histories) [20]. They reported inconsistent results regarding age, generally confirming our finding of no association between age and discrepancy [20]. As in our results, larger discrepancies were associated with an increasing number of medications [20]. Our study adds essential insights into how discrepancy varies across medication groups. In particular, we observed larger discrepancies in medications with variable usage, particularly ‘on-demand’ medications, which seems to be a new finding. The high ORs for discrepancy for respiratory, topically applied and ‘on-demand, personal’ medications - unlike cardiovascular, diabetes and daily used medications - may indicate that patients gradually adjust their medication usage to their individual needs. These behavioural adjustments by patients are often not communicated to the doctor or pharmacist- who will not always check the patient’s complete, and sometimes long, medication list - or pharmacist. Therefore, they are not recorded, or only by either the doctor or the pharmacist. This aligns with our results on the relatively high discrepancies where a medication was mentioned only by the patient, and the relatively high discrepancy in dosages.

Another systematic review reported other relevant factors associated with discrepancy, which we did not include in our research: the use of multiple outpatient pharmacies, the lack of a medication list, less experience of the admitting physician, patients’ insufficient understanding of their medications, and the reliance on a family member or caregiver as the consulted source instead of the patient [16]. We found less discrepancy between patients, GPs, or CPs regarding prescribed medications in patients living in a residential care home. Despite lower medication knowledge in this category, as observed in a previous study [19], this may be explained by the common use of MDD systems in residential care homes.

### Implications

#### Consequences of medication discrepancy 

Although there is much literature on patient adherence to prescribed medication - i.e. what patients ‘do wrong’ and how doctors and pharmacists could improve this - there is less attention to the more neutral description of the discrepancies between the medications prescribed by the GP, dispensed by the CP, and taken by the patient. Especially in patients with polypharmacy, a high degree of concordance between these three actors is essential for adequate medication management by GP and CP and for patient medication safety, i.e. avoiding overprescribing (duplicate or similar medications and high doses) and underprescribing (wrongly not prescribing or not increasing the dose). If doctors base their medical decisions (diagnostics, treatment) on incorrect information about medication, this may be detrimental to the patient’s health and may threaten patient safety. If doctors and pharmacists do not sufficiently check whether the medications they think the patient is taking match what the patient is actually taking, this unsafe situation will continue to exist.

#### Frequent medication review by health professionals 

Given the considerable discrepancies found in this and other studies [17, 21], we recommend that the medication list in the doctor’s or pharmacist’s computers and the medication the patient takes be routinely checked for every patient with polypharmacy who visits the GP or the pharmacy. Home visits provide a good opportunity to map out the patients’ actual medication use. The GP and CP can make so-called brown-bag assessments [11]. Medications not prescribed (or taken) on a fixed basis but variably – ‘on-demand’, ‘as needed’, ‘at the patient’s discretion’ – may need extra checks. Given our results, we also recommend paying special attention to respiratory and topical medications. Patients treated by various specialists [12] or receiving medication from multiple pharmacies [21] also deserve attention. Writing a referral letter and processing a specialist letter in the medical record are opportunities for the GP to review the medication list with the patient. The GP and pharmacist can discuss with the patient whether, if possible, they should choose one pharmacy and collect the medication prescribed by the specialist in the hospital from the community pharmacy. Clinical practice guidelines to improve medication management in patients with polypharmacy in primary care often recommend medication reviews performed by the GP or CP [5, 6, 22–26].

#### MDD systems 

We suggest discussing the advantages of Multidose Drug Dispensing (MDD) systems, which may reduce medication discrepancies in residential care homes, with all patients who use many medications. Martín-Oliveros and colleagues propose combining MDD with other elements, such as integrated care, medication review, and patient follow-up, to improve the management of patients with multimorbidity and polypharmacy [25]. An advantage of an MDD is its convenience for the patient and (informal) caregiver; the disadvantage can be reduced patient (and caregiver) knowledge of the medication [25]. There is a need for more knowledge about patients’ experiences with MDD systems. A Dutch study reported improvements in perceived medication safety and medication adherence, alongside problems with packaging opening and legibility [26].

#### Communication between healthcare professionals 

Discrepancies between GPs and CPs could be avoided by shared or at least seamlessly communicating electronic medication systems. Our results underscore the importance of collaboration between GPs, specialists, and pharmacists in the provision of medication to patients, as well as of the development of a national infrastructure for the transfer of medication data that supports this collaboration (for details, see Supplement, Box 5). Currently, as part of the “Kickstart medication transfer” programme, two regions in the Netherlands are developing a uniform national medication registration system, which combines registration of prescription, delivery, and administration data (Medication Process 9.0) [27]. Its results are expected by the end of 2026. Information technology that enables efficient medication reconciliation between healthcare providers will significantly reduce a major barrier to preventing and resolving discrepancies between physicians’ and pharmacists’ medication records: the need for time-consuming personal communication between healthcare providers, which, by its very nature, is often postponed or not taken at all.

#### Patient influence

In the future, ‘personal health environment’ apps - allowing patients to collect and manage their medical data - could play a role in the digital medical information infrastructure (see Supplement, Box 5). Already, patients increasingly have online access to their medical records, empowering them to participate actively in their disease management. The GP and patient can agree on which chronic medication - name, dose, and frequency - the patient can digitally request a repeat prescription. We recommend that patients be offered the option to signal when they notice that their medical records and reality do not match. Shared decision-making – involving the patient in exploring their experiences and treatment goals – is considered a prerequisite for effective and safe pharmacotherapy. This might also decrease discrepancies in prescribed medication between patients, GPs or CPs.

## Conclusion

We found an overall patient-GP-CP discrepancy on prescribed medication of 72% at the patient level and 22% at the medication level. There was less medication discrepancy between patients, GPs or CPs among patients living in residential care homes than among those living independently alone. Various medication characteristics, such as any additional medication and variable medication usage, increased the chance of a medication discrepancy between the patient, GP, or CP.

To minimise medication list discrepancies, we recommend routine and frequent medication reviews by GPs and CPs, greater patient involvement in medication monitoring, and better communication among healthcare professionals, including the integration of medical information systems to improve medication reconciliation. We emphasise that when collaborative, efficiency-enhancing information systems for medication registration are in place, multidisciplinary guidelines, regional interprofessional collaboration agreements, and the principle of shared decision-making should continue to determine the content of medication provision to patients.

## Supplementary Information

Supplementary Material 1.

Supplementary Material 2. STROBE Statement—Checklist of items that should be included in reports of crosssectional studies.

## Data Availability

Data are available upon reasonable request and can only be used in cooperation with the authors.

## Abbreviations

ATC: Anatomical Therapeutic Chemical classification system
CI: Confidence interval
CP: Community pharmacist
CYP2C9-inhibitors: Cytochrome P450 family 2 subfamily C member 9
GEE: Generalised estimating equation
GP: General practitioner
ICT: Information and communication technology
MDD: Multidose Drug Dispensing
N-criterion: Drug name-only criterion
NDF-criterion: Drug name-dose-frequency criterion
NSAID: Nonsteroidal anti-inflammatory drug
OR: Odds ratio
OTC: Over-the-counter
PIL: Polypharmacy Intervention Limburg
PPI: Proton pump inhibitors
SD: Standard deviation
SPSS: Statistical Package for the Social Sciences

## References

[1] Masnoon N, Shakib S, Kalisch-Ellett L, Caughey GE. What is polypharmacy? A systematic review of definitions. BMC Geriatr. 2017;17(1):230. 29017448 10.1186/s12877-017-0621-2 PMC5635569

[2] Eriksen CU, Kyriakidis S, Christensen LD, Jacobsen R, Laursen J, Christensen MB, et al. Medication-related experiences of patients with polypharmacy: a systematic review of qualitative studies. BMJ Open. 2020;10(9):036158. 10.1136/bmjopen-2019-036158 PMC7477975 32895268

[3] Davies LE, Spiers G, Kingston A, Todd A, Adamson J, Hanratty B. Adverse Outcomes of Polypharmacy in Older People: Systematic Review of Reviews. J Am Med Dir Assoc. 2020;21(2):181–7. 31926797 10.1016/j.jamda.2019.10.022

[4] Anderson LJ, Schnipper JL, Nuckols TK, Shane R, Sarkisian C, Le MM, et al. A systematic overview of systematic reviews evaluating interventions addressing polypharmacy. Am J Health-Syst Pharm AJHP Off J Am Soc Health-Syst Pharm. 2019;76(21):1777–87. 10.1093/ajhp/zxz196 PMC7170727 31612924

[5] Dutch College of General Practitioners. Multidisciplinaire richtlijn Polyfarmacie bij ouderen. Utrecht. 2012 Jan. Available from: https://richtlijnen.nhg.org//files/2021-02/2021-02-02%20%20Eindversie%20MDR%20Polyfarmacie.pdf

[6] Dutch College of General Practitioners. Multidisciplinaire Richtlijn Polyfarmacie bij ouderen.: Module Medicatiebeoordeling. Utrecht. 2019 Jan. Available from: https://www.nhg.org/sites/default/files/content/nhg_org/uploads/final_module_medicatiebeoordeling_2019.pdf

[7] Balon J, Thomas SA. Comparison of Hospital Admission Medication Lists With Primary Care Physician and Outpatient Pharmacy Lists. J Nurs Sch. 2011;43(3):292–300. 10.1111/j.1547-5069.2011.01409.x 21884375

[8] Olson MD, Tong GL, Steiner BD, Viera AJ, Ashkin E, Newton WP. Medication documentation in a primary care network serving North Carolina medicaid patients: results of a cross-sectional chart review. BMC Fam Pract. 2012;13(1):83. 22889327 10.1186/1471-2296-13-83 PMC3515456

[9] van den Bemt PMLA, van der Schrieck-de Loos EM, van der Linden C, Theeuwes AMLJ, Pol AG, Dutch, CBO WHO High 5s Study Group. Effect of medication reconciliation on unintentional medication discrepancies in acute hospital admissions of elderly adults: a multicenter study. J Am Geriatr Soc. 2013;61(8):1262–8. 23869999 10.1111/jgs.12380

[10] Rodríguez Vargas B, Delgado Silveira E, Iglesias Peinado I, Bermejo Vicedo T. Prevalence and risk factors for medication reconciliation errors during hospital admission in elderly patients. Int J Clin Pharm. 2016;38(5):1164–71. 27558355 10.1007/s11096-016-0348-8

[11] Bosch-Lenders D, Jansen J, Stoffers HEJHJ, Winkens B, Aretz K, Twellaar M et al. The Effect of a Comprehensive, Interdisciplinary Medication Review on Quality of Life and Medication Use in Community Dwelling Older People with Polypharmacy. J Clin Med. 2021;10(4). 10.3390/jcm10040600 PMC7915595 33562702

[12] Bosch-Lenders D, van den Akker M, Stoffers H, van der Kuy H, Schols J, Knottnerus JA. [How much do patients and health professionals (really) know? The surplus value of a home visit to the patient with polypharmacy by the practice nurse to support medication reviews in primary care]. Tijdschr Gerontol Geriatr. 2013;44:72–80. Wat weten de patiënt en zijn hulpverlener: de meerwaarde van een huisbezoek door de praktijkondersteuner bij de medicatiebeoordelingen van patiënten met polyfarmacie in de eerste lijn. 10.1007/s12439-013-0015-7 23508790

[13] Bosch-Lenders D. PIL:Polypharmacy Intervention Limburg: too much or too little? | Research with human participants. 2009 [cited 2024 Dec 3]. Available from: https://onderzoekmetmensen.nl/en/trial/32535

[14] Zorginstituut Nederland. Farmacotherapeutisch Kompas. Zorginstituut Nederland; [cited 2024 Dec 3]. Available from: https://www.farmacotherapeutischkompas.nl/

[15] ATCDDD - ATC/DDD Index. [cited 2024 Dec 3]. Available from: https://atcddd.fhi.no/atc_ddd_index/

[16] Saedder EA, Brock B, Nielsen LP, Bonnerup DK, Lisby M. Identifying high-risk medication: a systematic literature review. Eur J Clin Pharmacol. 2014;70(6):637–45. 24671697 10.1007/s00228-014-1668-z

[17] Flinterman LE, Batenburg R, Kenens RJ, Duijkers B. Huisartsen en praktijken in kaart: cijfers uit Nivel Beroepenregistraties in de Zorg 2023–2024. NIVEL; 2025 [cited 2026 Jan 10]. Available from: https://www.nivel.nl/nl/publicatie/huisartsen-en-praktijken-kaart-cijfers-uit-nivel-beroepenregistraties-de-zorg-2023-2024

[18] Josendal AV, Bergmo TS, Granas AG. Implementation of a shared medication list in primary care - a controlled pre-post study of medication discrepancies. BMC Health Serv Res. 2021;21(1):1335. 34903215 10.1186/s12913-021-07346-8 PMC8670071

[19] Hammar T, Mzil L, Eiermann B. Discrepancies in patients’ medication lists from pharmacies in Sweden: an interview study before the implementation of the Swedish National Medication List. Int J Clin Pharm. 2023;45(1):88–96. 36307661 10.1007/s11096-022-01480-x PMC9938824

[20] Hias J, Van Der Linden L, Spriet I, Vanbrabant P, Willems L, Tournoy J, et al. Predictors for unintentional medication reconciliation discrepancies in preadmission medication: a systematic review. Eur J Clin Pharmacol. 2017;73(11):1355–77. 28744584 10.1007/s00228-017-2308-1

[21] Heymans MW, Twisk JWR. Handling missing data in clinical research. J Clin Epidemiol. 2022;151:185–8. 36150546 10.1016/j.jclinepi.2022.08.016

[22] Leitliniengruppe Hessen D. S3-Leitlinie Multimedikation, Langfassung. 2. Auflage. AWMF-Registernummer: 053 – 043. 2021. Available from: https://www.awmf.org/uploads/tx_szleitlinien/053-043l_S3_Multimedikation_2021-08.pdf

[23] NICE Medicines and Prescribing Centre (UK). Medicines Optimisation: The safe and effective use of medicines to enable the best possible outcomes. 2015. Available from: https://www.nice.org.uk/guidance/ng5/evidence/full-guideline-6775454 26180890

[24] Pharmaceutical Society of Australia^. Guidelines for Comprehensive Management Reviews. Available from: https://www.ppaonline.com.au/wp-content/uploads/2020/04/PSA-Guidelines-for-Comprehensive-Medication-Management-Reviews.pdf

[25] Martín-Oliveros A, Plaza Zamora J, Monaco A, Anitua Iriarte J, Schlageter J, Ducinskiene D, et al. Multidose Drug Dispensing in Community Healthcare Settings for Patients With Multimorbidity and Polypharmacy. Inq J Med Care Organ Provis Financ. 2024;61:469580241274268. 10.1177/00469580241274268 PMC11526267 39373170

[26] Mertens BJ, Kwint HF, Van Marum RJ, Bouvy ML. Patients’ experiences with multidose drug dispensing: a cross sectional study. Int J Clin Pharm. 2019;41(1):104–12. 30478494 10.1007/s11096-018-0749-y PMC6394512

[27] Nictiz. Functional Design Medication Process 9 version 2.0.0 English version - informatiestandaarden. [cited 2025 Feb 5]. Available from: https://informatiestandaarden.nictiz.nl/wiki/mp:V2.0.0_Ontwerp_medicatieproces_9_ENG

