# Supplementary material for: Discrepancies in prescribed medications as reported by patients, general practitioners, and community pharmacists in older patients with polypharmacy in primary care
Source: BMC Prim Care. 2026 Apr 30;27:237. doi: 10.1186/s12875-026-03332-3 (PMC13277199; doi:10.1186/s12875-026-03332-3)
Supplement: Supplementary file 1 — Supplementary Material 1. [file 12875_2026_3332_MOESM1_ESM.docx]

**Discrepancies in prescribed medications as reported by patients, general practitioners, and community pharmacists in older patients with polypharmacy in primary care**

**SUPPLEMENT**

**Table S1-a. Over-the-counter (OTC) medicines (N=822): Pharmacological categories and patients (N=751) using at least one medicine in the described category**

| **OTC category** | **Medication level** | | **Patient level** | | **Medications/patient** | |
| --- | --- | --- | --- | --- | --- | --- |
|  | Number of OTC medicines | Percentage of all OTC medicines | Number of patients using ≥1 OTC medicine in the category | Percentage of all patients | Mean number of medicines per patient using ≥1 OTC med. | Range in number of medicines per patient |
| **All OTC medicines** | **822** | **100%** | **457** | **60.9%** | **1.8** | **1-6** |
| Paracetamol | 289 | 35.2% | 282 | 37.5% | N.A. | N.A. |
| Vitamins and dietary supplements | 211 | 25.7% | 157 | 20.9% | 1.3 | 1-5 |
| Musculoskeletal (external, topical) | 63 | 7.7% | 54 | 7.2% | 1.2 | 1-3 |
| Digestive tract | 51 | 6.2% | 45 | 6.0% | 1.1 | 1-3 |
| Dermatological | 45 | 5.5% | 35 | 4.7% | 1.3 | 1-2 |
| Ear-nose-throat and respiratory tract | 31 | 3.8% | 25 | 3.3% | 1.2 | 1-3 |
| Arthrosis and bone supplements | 29 | 3.5% | 27 | 3.6% | 1.1 | 1-2 |
| Vegetal and homeopathic products | 29 | 3.5% | 24 | 3.2% | 1.2 | 1-3 |
| Urinary tract | 22 | 2.7% | 22 | 2.9% | 1 | 1 |
| Psychotropic | 13 | 1.6% | 13 | 1.7% | 1 | 1 |
| NSAID’s | 10 | 1.2% | 9 | 1.2% | 1.1 | 1-2 |
| Other | 29 | 3.5% | 25 | 3.3% | 1.2 | 1-3 |
| **No OTC medicines** | **N.A.** | **N.A.** | **294** | **39.1%** | **0** | **0** |

**Table S1-b. Potentially dangerous over-the-counter (OTC) medicines in older patients with polypharmacy in primary care (N=751)**

| **OTC medicine** | **Medication level** | | **Patient level** | | |  |
| --- | --- | --- | --- | --- | --- | --- |
|  | Number of OTC medicines | Percentage of all OTC medicines | Number of patients using ≥1 OTC medicine | % of patients using ≥1 OTC medicine (n=457) | % of all patients (N=751) |  |
| **All** | **822** | **100.0%** | **457** | **100.0%** | **60.9%** |  |
| Potentially dangerous OTC medicines | 12 | 1.5% | 10 | 2.2% | 1.3% |  |
| - NSAIDs | 10 | 1.2% | 9 | 2.0% | 1.2% |  |
| - Antimycotics (CYP2C9-inhibitors, i.e. miconazole) | 2 | 0.2% | 1 | 0.2% | 0.1% |  |

**TABLE S2. Fixed and variable medication usage (N=751 patients, n=5977 prescribed medications)**

| **Prescribed medication category** | **Total number of medications per category** | **Fixed medication usage** | **Percentage fixed** | **Variable medication usage** | **Percentage variable** |
| --- | --- | --- | --- | --- | --- |
| Cardiovascular | 3197 | 3057 | 95.6% | 140 | 4.4% |
| Digestive tract | 504 | 11 | 2.2% | 493 | 97.8% |
| Diabetes | 424 | 303 | 71.5% | 121 | 28.5% |
| Respiratory tract | 386 | 5 | 1.3% | 381 | 98.7% |
| Analgesics | 245 | 7 | 2.9% | 238 | 97.1% |
| Psychotropic | 229 | 179 | 78.2% | 50 | 21.8% |
| Other-oral | 673 | 556 | 82.6% | 117 | 17.4% |
| Other-topical | 319 | 52 | 16.3% | 267 | 83.7% |
| **All*** | **5977** | **4170** | **69.8%** | **1807** | **30.2%** |
| Legend |  |  |  |  |  |
| * N=5989 prescribed medications. Injections given by the general practitioner (n=12) were excluded from the discrepancy analyses: Goserelin (n=4), testosterone (n=3), leuprorelin (n=2), teriparatide (n=2), gamma globulin (n=1). | | | | | |

**TABLE S3. Missing variables in discrepancy analyses**

| **Prescribed medication category** | **Total number of medications per category** | **Missing data** | **Percentage of missing data** | **Number of medications analysed per category** |
| --- | --- | --- | --- | --- |
| Cardiovascular | 3197 | 64 | 2.0% | 3133 |
| Digestive tract | 504 | 12 | 2.4% | 492 |
| Diabetes | 424 | 9 | 2.1% | 415 |
| Respiratory tract | 386 | 9 | 2.3% | 377 |
| Analgesics | 245 | 5 | 2.0% | 240 |
| Psychotropic | 229 | 7 | 3.1% | 222 |
| Other-oral | 673 | 21 | 3.1% | 652 |
| Other-topical | 319 | 8 | 2.5% | 311 |
| **All*** | **5977** | **135** | **2.3%** | **5842** |
| * Legend: see Table S2 | | | | |

**Table S4. Discrepancies between patients, general practitioners (GPs) or community pharmacists (CPs) for prescribed medication categories and criteria considering fixed or variable medication usage.**

| **Prescribed medication category** | **Number of medications per category** | **Discrepancy on medication name, dose, or frequency (NDF) (%)** | **Discrepancy on medication name, disregarding dose or frequency (N) (%)** | **Discrepancies using the NDF-criterion for fixed and the N-criterion for variable medication usage (%)** |
| --- | --- | --- | --- | --- |
| Cardiovascular | 3133 (100) | 484 (15.4) | 268 (8.6) | 476 (15.2) |
| Digestive tract | 492 (100) | 178 (36.2) | 112 (22.8) | 114 (23.2) |
| Diabetes | 415 (100) | 72 (17.3) | 44 (10.6) | 72 (17.3) |
| Respiratory tract | 377 (100) | 211 (56.0) | 129 (34.2) | 129 (34.2) |
| Analgesics | 240 (100) | 162 (67.5) | 106 (44.2) | 106 (44.2) |
| Psychotropic | 222 (100) | 87 (39.2) | 44 (19.8) | 75 (33.8) |
| Other-oral | 652 (100) | 196 (30.1) | 124 (19.0) | 180 (27.6) |
| Other-topical | 311 (100) | 194 (62.4) | 150 (48.2) | 155 (49.8) |
| **All*** | **5842 (100)** | **1584 (27.1)** | **977 (16.7)** | **1307 (22.3)** |
| * Legend: see Table S2 and S3 for excluded (n=12 injections) and missing data (n=135) in the discrepancy analyses. | | | | |

**Box S5. Description of the Dutch healthcare system regarding the prescription (doctors), delivery (pharmacists) and use (by patients) of medication, and developments therein since the PIL study data collection.**


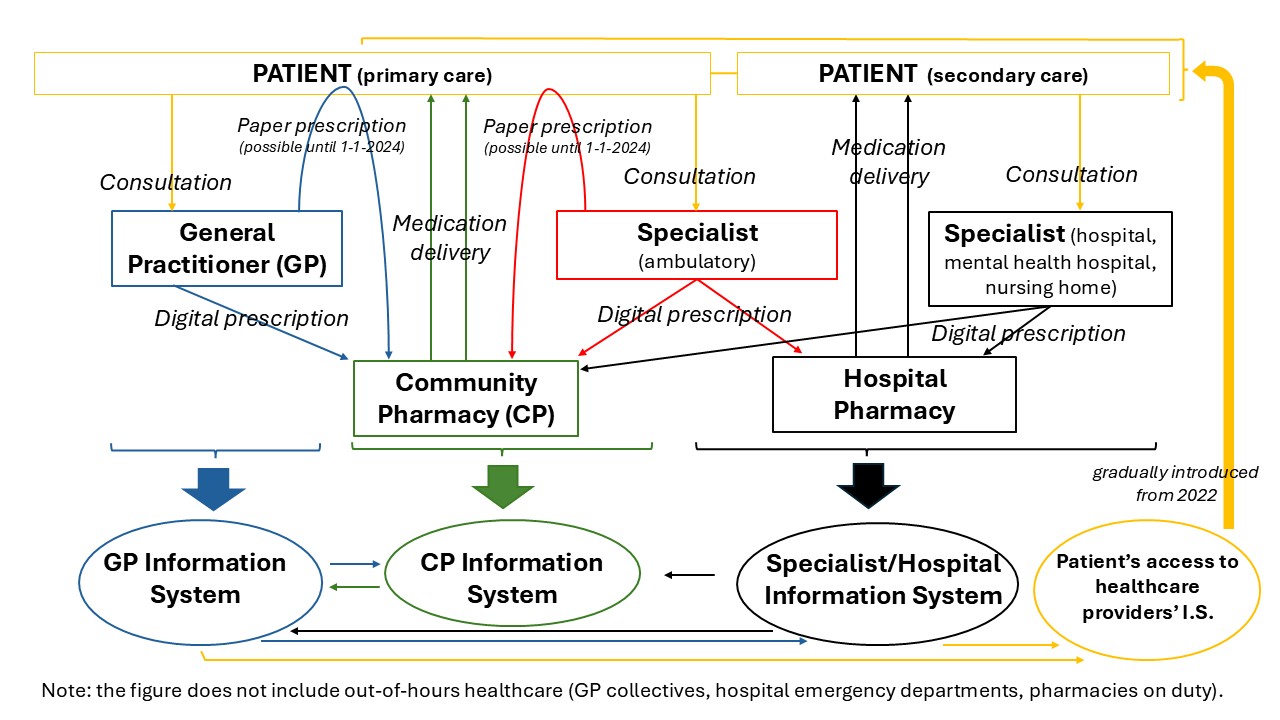


***Brief description of prescription and delivery of medications in the Netherlands (see Figure)***

Prescription and delivery

- Every citizen in the Netherlands is affiliated with a fixed GP (practice) and a fixed community pharmacy, both of their choice. GP practices usually deal with only a few pharmacies in the local or wider area. A pharmacy typically processes prescriptions from multiple GP practices and hospitals. Specialists working in an outpatient clinic usually prescribe to the patient's community pharmacy. In the hospital, prescriptions are sent to the hospital pharmacy. Upon discharge, the specialist forwards prescriptions to the hospital pharmacy or the patient's community pharmacy.
- Paper prescriptions (handwritten or printed) have gradually been phased out. Since January 2024, only digital prescriptions are allowed.
- Health insurers demand that doctors prescribe generic medications. Pharmacists should then dispense the least expensive medication (preferably a generic). Due to the variability in medication prices, this rule implies that a pharmacist may need to change the brand of a chronic medication every 3 months (i.e., the typical period for a chronic repeat prescription).

Chronic repeat prescriptions

- GP practices often have agreements with the most common pharmacy about chronic repeat prescriptions (a fixed number of repeats without doctor-patient contact, or an annual prescription). If there is close cooperation between the GP practice and the pharmacy (for example, within one health centre), the medication modules of the GP information system and the pharmacy information system are often linked. In case of multiple chronic medications (polypharmacy), an MDD can be considered, which requires a proper consultation (medication review) between the patient, GP, and pharmacy.
- Practices may offer patients the choice of ordering chronic repeat medication through the practice assistant (by phone or online), who prepares the prescriptions for approval by the GP.

Medication reconciliation

- GPs and community pharmacists participate in joint structured groupwise ‘pharmacotherapeutic consultation sessions’, for which Continuing Medical Education (CME) credits can be earned.
- In the Dutch healthcare system, general practitioners serve as gatekeepers to specialist care. GPs are expected to include the patient's current medication in referral letters to specialists. When patients are under long-term care by a specialist, the specialist manages changes (stopping, starting, or changing the dosage) to the medication(s) the specialist prescribes. This is ideally communicated to the GP in a specialist letter or discharge letter, and to the community pharmacy if the specialist sends the prescriptions directly to them. Another, less common, possibility is that the specialist informs the GP of the desired medication adjustments, and the GP handles implementing them.
- The medical Information systems of GPs and specialists are increasingly able to communicate with each other (documents and data transfer). Patients also increasingly have access to (view and download) their medical data from their GP and specialist, including their medication. Information systems for GPs and specialists contain medication modules that often communicate directly with the pharmacist's information system.

***Developments since the data collection for this study***

Our data were collected in 2010 and 2011. Since our data collection, we have identified developments in the Dutch healthcare system that could influence the prescription (by GPs), delivery (by community pharmacists), use (by patients), and communication about medication by the three actors.

Prescription and delivery

Shortly after completion of the data collection for the PIL study, the first version of the Multidisciplinary Guideline on Polypharmacy for Older People was published (2012); it was subsequently expanded over the years (1,2). We have no information on the implementation of these guidelines in the Netherlands. Despite a 'national agreement for primary care' for general practitioners, pharmacists and community nurses for the organization of care for chronic medication (3) and, very recently, a 'guideline for repeat medication' for GPs and pharmacists (4), there has been no development towards a (mandatory, reimbursed) structural collaboration between GPs and CPs regarding medication reviews. GPs continue to receive no compensation for the additional work in performing medication reviews. In contrast, pharmacists have been incentivised for medication reviews conducted as part of the Care for Older People module.

Software is now available for GP care that compares data from individual patient files with recommendations in clinical practice guidelines, including medication recommendations, thereby aiding GPs in their medication management, particularly in identifying possible medication errors (5,6). However, implementing this (expensive) software is optional for GPs. Moreover, identifying potential errors leads to extra tasks that must be addressed. As said, GPs are not reimbursed for the additional work that medication reviews entail.

Chronic repeat medications

An increase in the use of Multidose Drug Dispensing (MDD) systems (an automated dose-dispensing system in which the patient receives medication in labelled medication pouches on a medication roll) may have led to more coordinated medication registration between GPs and CPs and fewer discrepancies between GPs, CPs, and patients regarding medication. On the other hand, every change in MDD medication requires an additional procedure by the GP and the pharmacy, which often delays the correct entry of the medication change into the GP and pharmacy information systems.

Medication reconciliation

Concerning medication reconciliation, we mention several developments in information technology. Through the National Exchange Point (LSP; in its current format existing since 2015)(7), GPs and pharmacists can, after explicit patient consent, share a limited set of data — including prescribed medication in the past four months (GP data), and dispensed medication in the past six months (pharmacy data) — with other healthcare providers. The GP data are available to locum GPs (including GP out-of-hours clinics); the pharmacy data are available to (on duty) pharmacies (community and hospital), (locum) GPs, and medical specialists. The data remain stored at the respective sources: GPs and pharmacists. This infrastructure is particularly important for providing healthcare outside office hours.

Another development in information technology is the linkage of the pharmacy’s information system to the GP’s information system. This implies that prescriptions from specialists are immediately visible to the GP in his information system, so it is no longer necessary to manually copy this information from the specialist's letter. However, stopping specialist medication still requires human action. Moreover, a GP’s information system is not linked to all pharmacies’ information systems, implying that medication feedback is not received for all patients. Finally, hospital pharmacies are not linked to the information systems of GPs or community pharmacies.

However, with the development of the ‘law on digital information exchange in healthcare’ (entry into force 1 July 2023) (3,4), the Ministry of Health, Welfare and Sport has started a programme to improve digital medication transfer, based on the quality standard ‘Medication transfer’ (published in 2020) and the associated information standard (‘Medication process 9’, MP 9) (8,9). Implementation of this programme is evaluated and further developed in two regions of the Netherlands, where healthcare providers, pharmacies and patients collaborate in the so-called ‘kickstart medication transfer’ (10). Results are expected by the end of 2026. Only then can the implementation of a national medication transfer information structure be developed further.

In the future, ‘personal health environment’ apps - allowing patients to collect and manage their medical data - could play a role in this infrastructure (11). Currently (since the COVID-19 pandemic), patients have been allowed more access to their personal medical record(s) (GP, specialist), including medication. Some GPs offer patients the opportunity to reorder chronically used medication online. Theoretically, this allows patients to report assumed medication errors to their GP.

In summary, extensive guidelines on medication management (1-4) various legal measures, and various improvements in information technology to support the medication management of GPs (5,6,8,10), as well as in the collaboration of doctors and pharmacists on medication, have probably not led to substantial changes in the daily process of medication reconciliation over the past 15 years yet. A common digital medication prescribing and dispensing system for doctors and pharmacists is still under development. The influence of patients on medication management is still limited. A broader application of MDD systems may have decreased the GP-CP-patient discrepancies. Together, these developments may increase the chance that medication will be discussed between GPs, pharmacists, and patients. Therefore, discrepancies in medication between GPs, CPs, and patients may have decreased, but there is little reason to assume this change is substantial. After all, under our definition of GP-CP-patient discrepancies, discordance between two of the three actors is already considered a discrepancy. The recent attention given by the Dutch scientific journal "Huisarts en Wetenschap" to two 'tools' for improving collaboration between general practitioners and pharmacists in pharmaceutical care can be seen as a signal that there is still 'work to be done' (12).

***References Supplement***

1. Dutch College of General Practitioners. Multidisciplinaire richtlijn Polyfarmacie bij ouderen [Internet]. Utrecht; 2012 Jan. Available from: https://richtlijnen.nhg.org//files/2021-02/2021-02-02%20%20Eindversie%20MDR%20Polyfarmacie.pdf
2. Dutch College of General Practitioners. Multidisciplinaire Richtlijn Polyfarmacie bij ouderen.: Module Medicatiebeoordeling [Internet]. Utrecht; 2019 Jan. Available from: <https://www.nhg.org/sites/default/files/content/nhg_org/uploads/final_module_medicatiebeoordeling_2019.pdf>
3. Overheid.nl. Wet elektronische gegevensuitwisseling in de zorg [Law Electronic Data Exchange in Health Care]. [cited 2026 Jan 7]. Available from: <https://wetten.overheid.nl/BWBR0048095/2025-07-05>
4. Nictiz. Wegiz: Wet elektronische gegevensuitwisseling in de zorg [Wegiz: Law Electronic Data Exchange in Health Care]. [cited 2026 Jan 7]. Available from: <https://nictiz.nl/programmas/werken-aan-wegiz/>
5. NHG. NHGdoc. [cited 2026 Jan 13]. Available from: https://www.nhgdoc.nl/
6. VIPLive. VIPLive verbindt de zorg [VIPLive connects care]. [cited 2026 Jan 13]. Available from: https://viplive.nl/
7. AORTA-LSP. Betere zorg met de juiste informatie [Better care with the right information]. [cited 2026 Jan 7]. Available from: <https://www.aorta-lsp.nl/>
8. Nictiz. Functional Design Medication Process 9 version 2.0.0 English version - informatiestandaarden [Internet]. [cited 2025 Feb 5]. Available from: https://informatiestandaarden.nictiz.nl/wiki/mp:V2.0.0_Ontwerp_medicatieproces_9_ENG
9. Zorginstituut Nederland. Medicatieoverdracht - Overdracht van medicatiegegevens in de keten [Medication transfer – Transfer of medication information in the chain]. [cited 2026 Jan 7]. Available from: https://www.zorginzicht.nl/kwaliteitsstandaarden/medicatieoverdracht-overdracht-van-medicatiegegevens-in-de-keten
10. Samen voor Medicatieoverdracht. Medicatieoverdracht in de keten [Medication transfer in the chain]. [cited 2026 Jan 7]. Available from: https://www.samenvoormedicatieoverdracht.nl/kickstart-medicatieoverdracht/
11. PGO.nl. Met een PGO heb je jouw zorg in de hand [With a PGO [Personal Health Environment] you hold your care in your hand]. [cited 2026 Jan 7]. Available from: <https://www.pgo.nl/>
12. Beter samenwerken voor optimale farmaceutische zorg [Better collaboration for optimal pharmaceutical care]. Huisarts Wet 2025;68(9):48.
